# Plant Promoters and Terminators for High-Precision Bioengineering

**DOI:** 10.34133/bdr.0013

**Published:** 2023-07-07

**Authors:** Emily G. Brooks, Estefania Elorriaga, Yang Liu, James R. Duduit, Guoliang Yuan, Chung-Jui Tsai, Gerald A. Tuskan, Thomas G. Ranney, Xiaohan Yang, Wusheng Liu

**Affiliations:** ^1^ Department of Horticultural Science, North Carolina State University, Raleigh, NC 27607, USA.; ^2^ Biosciences Division, Oak Ridge National Laboratory, Oak Ridge, TN 37831, USA.; ^3^ The Center for Bioenergy Innovation, Oak Ridge National Laboratory, Oak Ridge, TN 37831, USA.; ^4^ Warnell School of Forestry and Natural Resource, University of Georgia, Athens, GA 30602, USA.; ^5^ Department of Plant Biology, University of Georgia, Athens, GA 30602, USA.; ^6^ Department of Genetics, University of Georgia, Athens, GA 30602, USA.; ^7^ Mountain Crop Improvement Lab, Department of Horticultural Science, Mountain Horticultural Crops Research and Extension Center, North Carolina State University, Mills River, NC 28759, USA.

## Abstract

High-precision bioengineering and synthetic biology require fine-tuning gene expression at both transcriptional and posttranscriptional levels. Gene transcription is tightly regulated by promoters and terminators. Promoters determine the timing, tissues and cells, and levels of the expression of genes. Terminators mediate transcription termination of genes and affect mRNA levels posttranscriptionally, e.g., the 3′-end processing, stability, translation efficiency, and nuclear to cytoplasmic export of mRNAs. The promoter and terminator combination affects gene expression. In the present article, we review the function and features of plant core promoters, proximal and distal promoters, and terminators, and their effects on and benchmarking strategies for regulating gene expression.

## Introduction

A plant’s DNA sequence can be fragmented into its smallest functional units to generate standardized, functionally interchangeable biological parts (bioparts) or biobricks. Bioparts are the fundamental building blocks in plant synthetic biology for assembling novel functional units or synthetic devices [1,2]. Bioparts include gene promoters, promoter *cis*-regulatory elements or motifs, exons, introns, terminators, and open reading frames (ORFs). Identification and characterization of individual bioparts and their respective biological interactions with other bioparts allows for improved understanding of transcription and gene expression as a whole. These bioparts are implemented in the design and construction of synthetic devices, which are integrated into plant (and nonplant) biological systems for precise regulation of gene transcription [3].

**Fig. 1.**
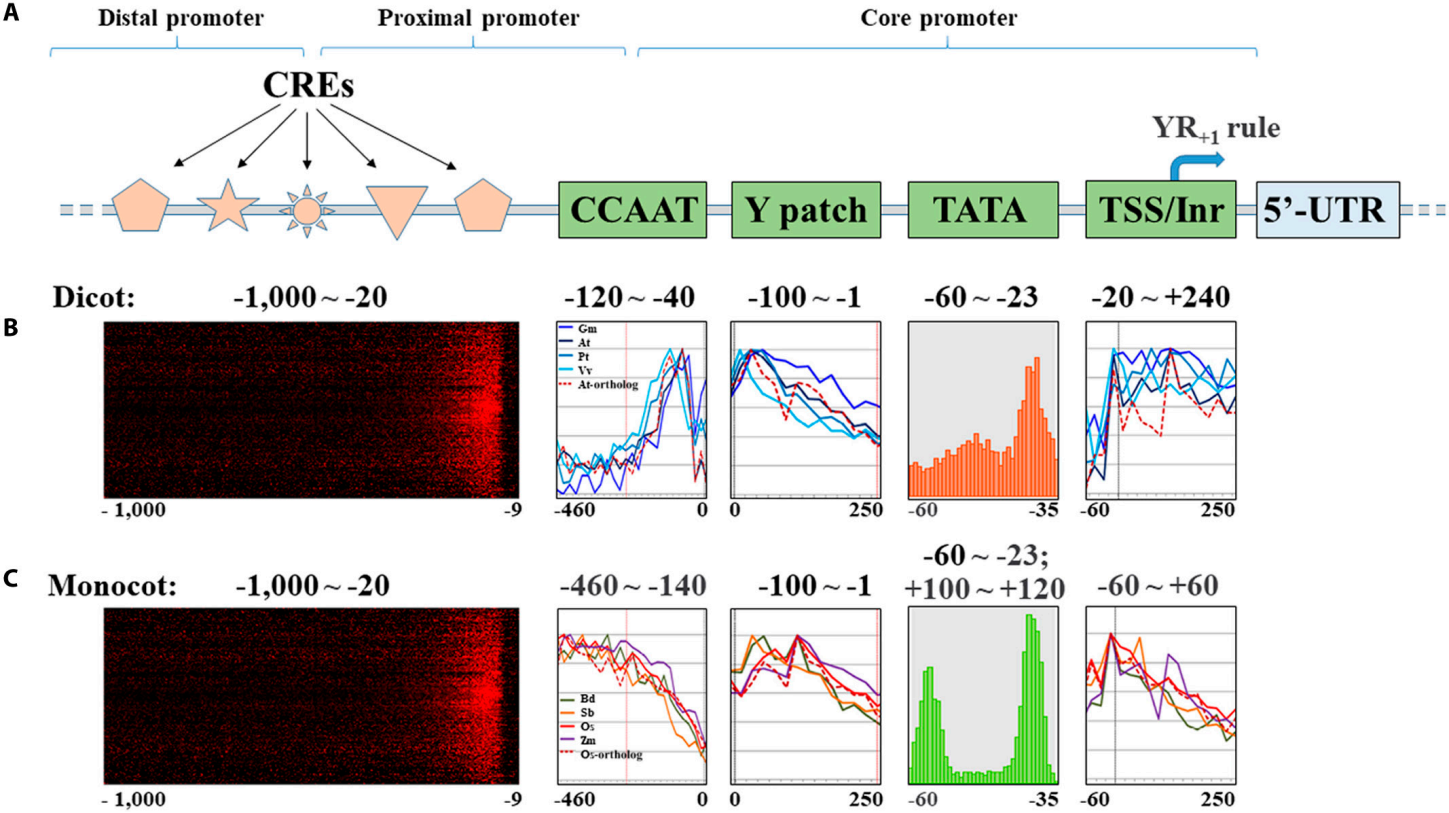
Locations of the *cis-*regulatory elements (CREs) and core promoter motifs CCAAT, Y patch, TATA-box, and TSS/Inr within dicot and monocot promoters. (A) Representative structure of a plant promoter. (B and C) Local distribution of short sequences (LDSS)-positive octamer CREs, normalized frequency distribution profiles of CCAAT, Y patch, and Inr, and the percentage of TATA-box-containing promoters at the indicated positions on the dicot (B) and monocot (C) promoters. The images of dicot and monocot CREs were adapted from [14] (*Arabidopsis*) and [13] (rice), respectively. The images of CCAAT, Y-patch, and Initiator (Inr) were adapted from [6] (soybean, *Arabidopsis*, poplar, grape, *Brachypodium distachyon*, sorghum, rice, and maize). The images of TATA-box were adapted from [5] (*Arabidopsis* and maize).

A plant gene promoter encompasses the DNA sequence flanking the transcription start site(s) (TSSs) of a gene that contains various promoter *cis*-regulatory elements, to which trans-acting transcription factors (TFs) bind and promote (or repress) initiation of gene transcription. Initiation of transcription is the first step for the designated genetic information of a synthetic device or system to be able to function as expected. Typically, a plant promoter spans the DNA region several hundred to a few thousand base pairs upstream of the TSS, with each promoter containing a core, a proximal, and a distal region based upon the function of the regulatory elements present in each region and their proximity to the TSS. The core promoter directly flanks the gene’s TSS and facilitates the assembly of a pre-initiation complex (PIC) upon the TSS(s), which consists of RNA polymerase (Pol) II and general transcription factors (GTFs). The PIC determines the basal transcription level of a gene, the direction of transcription, and the selection of TSSs, as one plant core promoter may harbor multiple and sometimes mutually exclusive TSSs [4]. The core promoter directs the assembly and recruitment of TFs through its *cis*-regulatory elements. A plant core promoter may contain conserved, direction-sensitive core promoter motifs such as the TATA-box (for TATA-box-containing promoters), downstream promoter element (DPE; for TATA-less promoters), initiator element (Inr), Y patch, CCAAT-box, and TSS(s) (Fig. 1). The TATA-box is named for its 8-base pair (bp) consensus sequence TATA(A/T)A(A/T)(A/G) and is commonly located 30 to 70 bp upstream of the TSSs in plants [5,6]. Binding of the TATA-binding protein (TBP), a subunit of the transcription factor IID (TFIID), to the TATA-box recruits RNA polymerase II (Pol II) to form the PIC and initiate transcription. In plants, most TATA-box-containing promoters are mainly involved in tissue-specific expression and stress responses [7,8]. Yamamoto et al. [9] found that the TATA-box-containing promoters in *Arabidopsis thaliana* tend to be associated with the presence of Y-patches and Inr elements, and have high promoter strength with sharp-peaked TSS clusters. In TATA-less promoters such as housekeeping photosynthesis-related gene promoters [7,8], a DPE with the consensus sequence RGWCGTG plays a similar function to that of the TATA-box for TFIID binding. DPEs are usually present approximately 30 bp downstream of the TSSs and are often found in plant promoters controlling stimulus-responsive genes [10]. The Inr element is another important core promoter motif directly covering TSSs [11] with a consensus sequence of PTCA_+1_NTPP, where A_+1_ is the initiator and the first base transcribed [11]. The TFIID binds to the Inr cooperatively with the TATA-box or the DPE to initiate recruitment of the PIC [12]. A Y patch is an 8-bp motif consisting mostly of pyrimidine C and T dimers (CT or TC), located 1 to 100 bp upstream of the TSSs, and has a distribution peak around the TSSs [13]. The CCAAT-box is the binding site for the CCAAT-binding factor (CBF) nuclear transcription factor Y (NF-Y). The binding of NF-Y to the CCAAT-box can result in positive or negative posttranslational histone modifications, contributing to activation or repression of gene expression [15]. Regarding the sequences surrounding the TSSs, Yamamoto et al. [13] reported a “YR Rule,” indicating that there is usually a C or T nucleotide 1 bp upstream of the TSSs and an A or G nucleotide 1 bp downstream of the TSSs (i.e., Y, C_−1_ or T_−1_; R, A_+1_ or G_+1_) for both *Arabidopsis* and rice.

The basal transcription level conferred by core promoters can be greatly increased (or inhibited) by enhancers (or repressors/silencers), which are promoter motifs located upstream of the core promoter regions and define the proximal and distal promoter regions (note: enhancers and repressors can also be located downstream of the TSSs). *Cis*-regulatory elements interact bidirectionally and in tandem with TFs, cofactors, and chromatin-remodeling complexes to determine the strength and the temporal and spatial expression patterns of a gene during plant growth and development and in response to changing environmental conditions. Studies in which promoters were functionally analyzed for constitutive, tissue-specific, or inducible expression have been extensively reviewed recently [10,16–22] and are not part of this review. *Cis*-regulatory elements used for plant synthetic promoter engineering have also been reviewed recently [23–28].

The proper combination of a promoter and a terminator is essential for successful gene transcription as the processing of the 3′-end of an RNA transcript is the last essential step in mRNA biogenesis. The terminator is one of the 3′-end regulatory elements and marks the end of an RNA transcript. It assists transcription initiation by interacting with TFs and the C-terminal domain of Pol II, and halts transcription by adding termination signals on the newly synthesized transcript, triggering the release of the transcript from the transcription machinery. Proper mRNA termination promotes the transport of mRNAs from the nucleus to the cytoplasm [29,30] and stabilizes and protects the mRNAs from degradation [31–35]. Thus, proper mRNA termination is necessary for the translation of mRNAs into proteins [31,35–37]. Proper mRNA termination also prevents read-through transcription of downstream sequences [38,39]. Termination factors interact with many RNA processing and degradation enzymes, which in turn define the fate and half-life of the transcripts. There are 2 termination pathways, i.e., transcription termination of protein coding transcripts (i.e., mRNAs) and transcription termination of noncoding RNAs (ncRNAs). Termination of mRNAs leads to the production of stable RNA transcripts that are transported to the cytoplasm, while termination of ncRNAs leads to their sequestration in the nucleus and subsequent degradation. In this review, we will only discuss mRNA transcriptional termination and its relevance for synthetic biology.

Thus, plant core promoters, proximal and distal promoters, and terminators are the starting bioparts regulating gene expression, and it is of crucial importance to determine the best promoter and terminator combination for highly efficient transcription of transgenes in high-precision plant bioengineering.

## Plant Core Promoters

Generally, dicot and monocot core promoters perform well when used in respective dicot and monocot species [5]. Core promoter performance is attributed to the nucleotide sequence, position, and number of copies of each core promoter motif, as well as the core promoter’s chromatin configuration. It was reported that 32%, 19%, and 38% genes in *Arabidopsis* [7], rice (*Oryza sativa*) [8], and maize (*Zea mays*) [40], respectively, are TATA-box-containing genes. Kumari and Ware [6] conducted an in silico genome-wide analysis of core promoter motifs in the dicot species *Arabidopsis*, soybean (*Glycine max*), poplar (*Populus trichocarpa*), and grape (*Vitis vinifera*), and in the monocot species rice, maize, sorghum (*Sorghum bicolor*), and purple false brome (*Brachypodium distachyon*). They found that the Inr motifs were located −20 to +240 bp upstream of the TSSs of the promoters in the 4 dicot species, while for the promoters of the 4 monocot species the Inr motifs stretched from −60 to +60 bp and from +100 to +120 bp, suggesting a distinct difference in the genome-wide distribution of the motif between dicot and monocot species. Similarly, the CCAAT-box was mainly positioned −120 to −40 bp in dicot promoters but positioned −460 to −140 bp in monocots [6]. Additionally, it was reported that less than 18% of *Arabidopsis* promoters and 50% of rice promoters contain Y patches [8,41]. All these distinctions indicate the differences in the functions of the core promoters between monocots and dicots. Thus, it is reasonable to use dicot and monocot core promoters for synthetic promoter engineering in respective species types.

To date, the most widely used core promoter is the minimal CaMV *35S* promoter, which has shown functional expression in nearly all dicot species and one monocot species, i.e., rice [42,43] (see Table 1 for a list of representative plant core promoters used for gene expression). While plant core promoter engineering is still in its infancy, a pivotal study was recently published describing plant core promoter analysis and engineering [5]. Jores et al. [5] constructed individual core promoter libraries from *Arabidopsis*, maize, and sorghum genomes. These libraries included the core promoter regions −165 to +5 nucleotides (nt) (relative to the TSSs) of 18,329, 34,415, and 27,094 protein-encoding and microRNA genes from each of the respective species. Evaluation of the core promoters using tobacco leaf agroinfiltration and maize leaf protoplast expression revealed substantial differences in core promoter strength, indicating that dicot and monocot plants interact with the same core promoter in different manners. Interestingly, core promoter strength was positively associated with gene expression levels only in some of the genes. GC content of the core promoters showed negative correlation with core promoter strength in the tobacco leaf expression system but had no correlation in the maize protoplast expression system. This finding could be attributed to the differences between the GC content of the promoters in these species because tobacco and *Arabidopsis* promoters are AT-rich, while maize and sorghum promoters tend to be higher in GC content. Moreover, the distribution locations of TATA-box have one peak ~30 bp upstream of the *Arabidopsis* TSSs, but 2 or 3 peaks upstream of the sorghum (~30 and ~40 bp) and maize (~30, ~55, and ~70 bp) TSSs. They also noticed that maize promoters with TATA-boxes in one of the 3 peaks or closer to the TSSs have higher strength than those with TATA-boxes elsewhere. In addition, TATA-box-containing core promoters exhibited up to 4-fold higher strength than TATA-less core promoters, and interestingly, the presence of Inr elements and Y patches was linked with significantly increased core promoter strength in *Arabidopsis*, maize, and sorghum [5].

**Table 1.** List of representative plant core promoter elements used for gene expression.

Core promoter element	Consensus sequence	Approximate location	Reference
TATA-box	TATAWAW	−70 to −20 bp	[5,6]
Inr	YYA_(+1)_NWYY	Overlapping +1	[44]
Y patch	CYTCYYCCYC	+20 to +80	[13]
TC motif	YYYYYY	−39 to −26	[41]
GA element	RRRRRRRR	+25 bp to +75 bp	[9]
CA element	YMMMMMMM	+1 bp to +30 bp	[9]
GC-box	GGGCGG	−70 bp to +250 bp	[6]
CAAC	MCMAMCCM	−50 bp to +40 bp	[9]
DPE	RGWCGTG	+30 bp	[10]
CCAAT-box	CCAAT	−80 bp	[10,15]

More importantly, Jores et al. [5] conducted plant core promoter engineering by generating random core promoters with average nucleotide frequencies similar to those of *Arabidopsis* or maize, followed by the addition of TATA-box, Inr, and/or Y patch motifs. As expected, the randomized core promoters had very weak strength, but the addition of the 3 motifs significantly increased the random core promoter activities individually or in combination, with TATA-box and Inr increasing the strength the most and the least, respectively. The strongest synthetic core promoters they developed had strength comparable to the minimal *35S* promoter. These results demonstrate that rational design and construction of plant synthetic core promoters with varying strength can be achieved by inserting core promoter motifs into an appropriate nucleotide background. Based on these results, computational models were developed for in silico evolution of synthetic core promoters to predict and improve core promoter strength. The models showed that the developed synthetic core promoters could work independent of the choices of enhancers [5]. Additionally, Jores et al. [5] found that inserting a TATA-box into the previously TATA-less core promoters of *Arabidopsis*, maize, and sorghum enhanced the core promoter’s strength. This is different from the finding by Nakamura et al. [11], which mutated the TATA-less Inr-containing core promoter of the tobacco *psaDb* gene to then contain a TATA-box without Inr, and found that the Inr rather than the TATA-box contributed to light-responsive transcriptional regulation of the photosynthesis gene.

It was reported that active (constitutive or housekeeping) core promoters are featured by the presence of nucleosome depletion regions (NDRs) since their nucleosomes are highly dynamic, ensuring the accessibility of the transcription machinery [45,46]. It was also reported that the NDRs in the active core promoters in *Arabidopsis*, rice, sorghum, and maize have high G/C content [46–48]. Srivastava et al. [49] identified that mutations in TATA-boxes or Inr elements of the *Arabidopsis* light-regulated promoters could result in the formation of nucleosomal structures, inhibiting gene expression. Oldfield et al. [50] found that the histone-fold domain protein NF-Y maintains the core promoter region in a nucleosome-depleted state in metazoans. Oldfield et al. [50] also found that loss of NF-Y binding to the core promoters disrupts the core promoter’s chromatin architecture, resulting in nucleosome enrichment on the core promoters and repression of gene expression. NF-Y is a ubiquitously expressed, heterotrimeric TF and the key player of TSS selection. Binding of NF-Y to its binding site CCAAT in the *Flowering Locus T* (*FT*) gene promoter in *Arabidopsis* and rice activated *FT* expression [51]. Still, the key differences in the chromatin landscapes of the dicot and monocot core promoters remain largely unknown.

It is worthwhile to point out that the practical use of plant promoters for gene expression almost always includes the use of 5′-untranslated regions (UTRs) and leader introns that are located within 5′-UTRs since they affect mRNA levels posttranscriptionally [50] and gene function in a length-dependent manner [52]. The average length of 5′-UTRs is 155 and 259 bp in *Arabidopsis* and rice, respectively [49]. It was reported that the length, GC content, and leader intron number of a 5′-UTR showed significant positive correlation with the expression breadth in various tissues of *Arabidopsis*, rice, maize, and sorghum [46]. It was also reported that the presence of leader introns enhanced gene expression. Examples include the leader introns of the *Arabidopsis ubiquitin3/10/11* (*UBQ3*, *UBQ10*, and *UBQ11*) [53], *Mg^2+^/H^+^ exchanger* (*MHX*) [54], and *cytochrome c oxidase subunit 5c* (*COX5c*) [55], tobacco *Ubiquitin.U4* (*Ubi.U4*) [56] and *carnation S-adenosylmethionine decarboxylase9(CSDC9)* [57], tomato *(Solanum lycopersicum)ascorbate peroxidase20* (*APX20*) [58], potato *(Solanum tuberosum)sucrose synthase3* and *4* (*Sus3*; *Sus4*) [59,60], mustard (*Brassica juncea*) *S*-*adenosylmethionine decarboxylase2* (*SAMDC2*) [61], rice *β-tubulin isotype 6* (*Ostub6*) [60,62] and *16* (*Ostub6*) [63], and *ubiquitin3* (*rubi3*) [64]. Leader introns may contain *cis*-regulatory elements [65] and affect transcription, mRNA stability and export, and tissue-specific gene expression [53,66–74].

## Plant Proximal and Distal Promoters

Plant natural promoters contain numerous *cis*-regulatory elements widely distributed across their proximal and distal promoter regions, with many of them acting as specific regulators of gene expression, i.e., stimulating (enhancers) or repressing (repressors) the basal expression levels conferred by the core promoters. Enhancers and repressors are direction-insensitive and are not typically position-restricted, increasing the likelihood of functioning in both dicot and monocot systems. It was found that about 30 to 50% of 8-bp promoter motifs are conserved between *Arabidopsis* and rice [13], indicating that transcriptional regulation is relatively conserved between dicots and monocots. As demonstrated by Belcher et al. [75], *cis*-regulatory elements from a different source (yeast in this case) could be used together with a plant core promoter to develop functional synthetic promoters that work well with plant endogenous transcriptional machinery. Screening of a synthetic promoter library consisting of 5 *cis*-regulatory elements from yeast and 5 plant core promoters showed that each *cis*-regulatory element has an independent effect on promoter strength, indicating the orthogonality of the *cis*-regulatory elements in engineering of synthetic promoters with tunable expression levels in a heterologous plant system.

Various native plant promoters have been functionally characterized and used for constitutive or conditional transgene expression (Table 2; also see the listed plant native promoters in [16,18–22]). Plant promoter databases have also been created for the annotation and curation of the information about functionally active promoters from various plant species. These include PlantProm (http://linux1.softberry.com/berry.phtml?topic=plantprom&group=data&subgroup=plantprom) [117], TransGene Promoters (TGP; http://wwwmgs.bionet.nsc.ru/mgs/dbases/tgp/home.html) [118], Plant Promoter Database (PPDB; https://ppdb.agr.gifu-u.ac.jp/ppdb/cgi-bin/index.cgi#about) [119,120], and Root-associated Genes and Promoters Database (RGPDB; http://sysbio.unl.edu/RGPDB/) [121].

**Table 2.** List of representative plant promoters used for gene expression.

Promoter	Source	Transgenic plant	Expression pattern	References
Constitutive promoters				
*Act2*	*A. thaliana*	*A. thaliana*	Constitutive	[76]
*Act-1*	*O. sativa*	*O. sativa*	Constitutive	[77]
*UBQ1*	*A. thaliana*	*N. tabacum*	Constitutive	[78]
*Ubi1, Ubi2*	*Panicum virgatum*	*P. virgatum; O. sativa; N. tabacum*	Constitutive	[79]
*Ubi1*	*Zea mays*	*Z. mays*	Constitutive	[80,81]
Tissue-specific promoters				
*SlREO*	*S. lycopersicum*	*S. lycopersicum*	Root	[82]
*NAC10*	*O. sativa*	*O. sativa*	Root	[83]
*PAT21*	*S. tuberosum*	*S. tuberosum*	Tuber	[84]
*hspr*	*A. thaliana*	*A. thaliana*	Vascular tissue	[85]
*Pfn2*	*P. virgatum*	*O. sativa*	Vascular tissue	[86]
*PEPC*	*Z. mays*	*Z. mays*	Leaf	[87]
*Lhcb*	*P. virgatum*	*O. sativa*	Green tissue	[88]
*TA29*	*N. tabacum*	*N. tabacum*	Flower	[89]
*Lat52*	*S. lycopersicum*	*N. tabacum*	Pollen	[90]
*Zm13*	*Z. mays*	*Tradescantia paludosa*; *Z. mays*	Pollen	[91]
*Oleosin*	*A. thaliana*	*G. max*	Seed	[92]
*Glutenin*	*T. aestivum*	*T. aestivum*	Seed	[93]
*D-hordein*	*T. aestivum*	*Z. mays*	Seed (endosperm)	[94]
*E8*	*S. lycopersicum*	*S. lycopersicum*	Fruit	[95]
Abiotic stress-inducible promoters				
*Adh-1*	*Z. mays*	*N. tabacum*	Anaerobic conditions	[96]
*wun1*	*S. tuberosum*	*N. tabacum*	Wounding	[97]
*GBSS*	*S. tuberosum*	*S. tuberosum*	Sugar	[98]
*HSP18.2*	*A. thaliana*	*A. thaliana*	Heat shock	[99]
*Rd29*	*A. thaliana*	*A. thaliana; N. tabacum; O. sativa*	Drought, cold, salt	[100]
*SR2*	*Phaseolus vulgaris*	*N. tabacum*	Heavy metals	[101]
*CCA1*	*A. thaliana*	*A. thaliana*	Light cycle	[102]
*UGT71C5*	*A. thaliana*	*A. thaliana*	Light	[103]
*GSE*	*O. rufipogon*	*A. thaliana*	Light	[104]
Biotic stress-inducible promoters				
*win3.12*	*Populus sp. hybrid*	*S. tuberosum*	*Fusarium solani*	[105]
*R2329*	*O. sativa*	*O. sativa*	*Magnaporthe grisea*	[106]
*Bs3*	*Capsicum annuum*	*N. benthamiana*	*Xanthomonas campestris pv. vesicatoria*	[107]
*CaPrx*	*Coffea arabica*	*N. tabacum*	*Meloidogyne incognita*	[108]
*4×M1.1, 4×M2.3*	*G. max*	*G. max*	*Heterodera glycines*	[107, 109]
*IFS2*	*G. max*	*G. max*	*Bradyrhizobium japonicum*	[110]
*SAG12*	*A. thaliana*	*S. lycopersicum*	Senescence	[111]
*SEOF1*	*Pisum sativum*	*N. tabacum*	Methyl jasmonate, auxin, abscisic acid (ABA)	[112]
*Em*	*T. aestivum*	*O. sativa*	ABA	[113]
*Rd29*	*A. thaliana*	*A. thaliana, N. tabacum*	ABA	[114]
*SAUR15A*	*G. max*	*N. tabacum*	Auxin	[115]
*Chn48*	*N. tabacum*	*N. tabacum*	Ethylene	[116]

The strength and expression patterns of plant native promoters are determined by the presence of combinatorial *cis*-regulatory elements and their 3-dimensional structures following the binding of their respective TFs [122,123]. Although reports have been published for the 3-dimensional structures of promoters from *Escherichia coli* [124,125], *Xenopus tropicalis* [126], and *Homo sapiens* [127,128], 3-dimensional structures have not been constructed for any plant promoters. Fortunately, *cis*-regulatory elements can be taken out of their natural context and be implemented as bioparts for synthetic promoter engineering. Known *cis*-regulatory elements can be identified in a plant promoter by searching against the 3 promoter motif databases PlantCARE [129], PLACE [130], and TRANSFAC [131], while novel *cis*-regulatory elements can be discovered through promoter serial deletion and de novo motif discovery [26]. It was shown that bioinformatics tools developed for de novo motif discovery have limited prediction efficiency (15 to 25%) of true motifs [132,133], thus making experimental function validation necessary. Liu et al. [134] developed an ensemble approach by focusing on overlapping motif regions detected by multiple de novo motif discovery tools, which improved the prediction efficiency to be 68.8% and led to the discovery of 23 experimentally validated, novel soybean cyst nematode (SCN)-inducible motifs in soybean. Using the same approach, Yang et al. [135,136] identified well-conserved salt- or drought-responsive promoter motifs in hybrid poplar, which were used to generate functionally validated water-deficit stress-, salt stress-, and osmotic stress-inducible synthetic promoters.

**Fig. 2.**
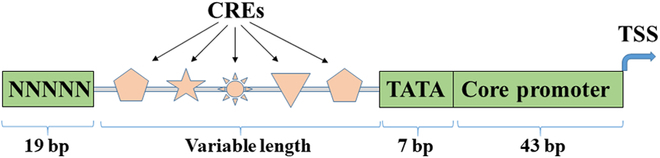
Schematic design of the synthetic promoters by Cai et al. [137], which contain a 19-bp random sequence (NNNNN), a region of variable length to which CREs are added, a TATA-box, and a 43-bp core sequence plus a transcription start site (TSS).

With the availability of functionally characterized *cis*-regulatory elements, it still remains largely undetermined how to rationally and reliably engineer plant synthetic promoters. To date, most plant synthetic promoter engineering studies have implemented a trial-and-error approach by using multiple copies of one or multiple motifs in front of a core promoter. Recently, Cai et al. [137] established a design system for plant synthetic promoter engineering by inserting various *cis*-regulatory elements between a 19-bp random nucleotide sequence and a TATA-box motif (TATATAA) in front of a 43-bp-long core promoter region and a TSS (Fig. 2). They found that random combinations of different trimerized *cis*-regulatory elements gave rise to increases in synthetic promoter strength than individual trimers. They also found that changes to the flanking sequences or relative positions of the *cis*-regulatory elements and moderate increases in spacing contributed negligible effects to promoter strength, implying that the corresponding TFs may work relatively independently other than via direct protein-protein interactions. However, they also reported that the >50-bp distance between the first motif at the 5′-end and the TATA-box significantly affected promoter strength. This raises an interesting nuance regarding motif spacing. The short spacing limit identified here may be pertinent to the specific motifs tested but may not be applicable to other classes of motifs, e.g., stress-inducible motifs that may function in a much less distance-restricted manner [138].

Although it remains largely unknown how fine-tuning gene expression is achieved via a combination of *cis*-regulatory elements, machine learning algorithms could be applied to build models for predicting synthetic promoter expression patterns by using multiple features, e.g., the presence and absence, location, copy number, and combinatorial relationships of *cis*-regulatory elements, as input variables. *Cis*-regulatory information has been implemented to predict biotic and abiotic stress- [139], high salinity- [140], and iron deficiency-responsive [141] transcription of *Arabidopsis* native promoters. Zou et al. [139] found that the prediction models based on combinatorial relationships (obtained by using a classification algorithm to integrate association rule mining) performed significantly better than those based on only singular traits, i.e., copy number or location.

## Plant Terminators

A gene’s terminator sequence mediates transcriptional termination, 3′-end processing, stability, translation efficiency, and nuclear to cytoplasmic export of the mRNA transcripts [142]. A terminator may contain one or more polyadenylation signals (PASs; with consensus sequence of AAUAAA) in the 3′-UTR. Once the poly-A signals are transcribed, a cleavage and polyadenylation specificity factor (CPSF) recognizes the PAS(s) and cleaves the pre-mRNA 10 to 30 bp downstream of the PAS, freeing the mRNA from the Pol II transcription machinery [143]. After the cleavage, a poly(A) tract of ~200 nt in length is added to the mRNA at the cleavage site (CS). Improper termination or unpolyadenylated mRNA transcripts serve as the templates for RNA-dependent RNA polymerase 6 (RDR6) to generate small interfering RNAs (siRNAs), leading to posttranscriptional gene silencing (PTGS) due to homology-based mRNA cleavage or translational repression [144,145].

In plants, 3 *cis*-termination elements function cooperatively to find the correct PAS(s) in the 3′-UTRs and initiate transcriptional termination. The 3 *cis*-termination elements include the near upstream element (NUE; AAUAAA-like motifs), the far upstream element (FUE), and the CS [146–149]. NUEs are short (6 to 10 bp) A-rich sequence located approximately 10 to 40 nt upstream of the poly(A) sites. FUEs are also short (6 to 18 bp) sequence with a more diverse nucleotide enrichment (U>A>G) and are typically found starting 30 bp upstream of the poly(A) sites. FUEs enhance processing efficiency at the CSs. The CS is a dinucleotide (CA or UA) typically located within the FUE where polyadenylation takes place. Less than 10% of terminators in *Arabidopsis* [146,150] and rice [151] contain the canonical PAS (AAUAAA). Genes can also contain multiple PASs [152].

Evidence shows that transgene expression level is significantly affected by the chosen terminators. In *Arabidopsis*, Pérez-González and Caro [153] demonstrated a comparison between the *Arabidopsis heat shock protein 18.2* terminator (*tHSP18*) and the *35S* terminator (*t35S*). The *tHSP18*, when used with the firefly luciferase (LUC) reporter gene driven by the *35S* promoter (*35S:LUC*), showed significantly higher protein levels and lower rates of promoter DNA methylation in transgenic *Arabidopsis* plants than the *t35S*. Using various promoters, de Felippes et al. [154] observed higher green fluorescent protein (GFP) fluorescence in *Agrobacterium*-infiltrated tobacco leaves when the *Arabidopsis tHSP18* was used, followed by the *A. tumefaciens Nopaline synthase* terminator (*tNos*), and then the *Arabidopsis RuBisCO small subunit* terminator (*tRbcS*). Differences in selection accuracy between terminator CSs may contribute to the differences in trans(gene) expression by various terminators. de Felippes et al. [154] found that only 40% of the transcripts generated from the overexpressed *GFP* transgene with *tRbcS* had the same poly(A) sites, while 76% of the *GFP* transcripts with *tHSP18* were cleaved at the same locations.

Terminators isolated from exogenous sources such as *tNos*, *A. tumefaciens Octopine synthase* terminator (*tOcs*), and *t35S* are frequently used in plant transgenic experiments. However, evidence shows that the terminator sequences from plant endogenous genes have shown even greater potential to increase transgene expression than exogenous terminators [155] (Table 3). Production of the miraculin (MIR) protein by the *35S* promoter was greatly increased (6.5×) in transgenic tomato when the tomato *tHSP18* was used over *tNos* [159]. Similarly, the native *MIR* terminator showed significantly higher MIR production in transgenic tomato than *tNos* when the *MIR* gene was driven by the *35S* promoter or the native or a sequence-optimized *MIR* promoter [165]. Diamos and Mason [156] compared the expression levels of *35S:GFP* using one of 20 native terminator sequences from various plant species and found that, in most cases, plant native terminators led to higher GFP protein production than *t35S* or *tNos*. Moreover, these plant native terminators showed variable abilities in regulating gene expression. Ingelbrecht et al. [166] studied the effects of a handful of tobacco native terminator sequences, including the terminators from a *2S* seed storage protein gene, an *RbcS* gene, an extensin gene, and a chalcone synthase gene on transcription of the *neomycin phosphotransferase II* (*nptII*) gene driven by the *35S* promoter (*35S:nptII*). They found that *tRbcS* led to the highest mRNA accumulation and protein production, corresponding to 3, 5, 10, and 60 times that of the *Ocs*, *2S*, extensin, and chalcone synthase terminators, respectively. These results suggest that terminators play an important role in posttranscriptional processes including the 3′-end processing efficiency and/or mRNA stability. Interestingly, Nagaya et al. [152] found that *Arabidopsis tHSP18* always procured the highest mRNA and protein abundance of the *Renilla* and *firefly luciferase* (*Rluc* and *Fluc*) reporter genes when driven by the *35S* promoter in both *Arabidopsis* and rice protoplasts, indicating that a plant native terminator could function well in both dicot and monocot species.

**Table 3.** List of representative plant terminators used for gene expression.

Terminator	Terminator source	Length (bp)	Effect ^a^	Transformed species	Expression system	References
*tACT3*	*N. benthamiana*	617	3.9× higher GFP than with *tNOS*	*N. benthamiana*	Transient ^b^	[156]
		1,044	~7.5× higher GFP than with *tNOS*	*Lactuca sativa*		
*tACT3* *–tRb7MAR*	*N. benthamiana (tACT3); N. tabacum (tRb7; tTM6)*	2,213	~8× higher GFP than with *tNOS*	*N. benthamiana*	Transient ^b^	[156]
*tACT3* *–tTM6MAR*		2,243	~7× higher GFP than with *tNOS*	*N. benthamiana*	Transient ^b^	[156]
*tEU*	*N. tabacum*	732	2.5× higher *GFP* mRNA and 2.5× higher GFP protein than with *tVspB3* (*Tobacco etch virus (TEV)* promoter)	*N. benthamiana*	Transient ^b^	[157]
		1,900	~1.5×higher DsRed than with *tNOS*;~44× higher DsRed than with *tNOS*	*N. benthamiana*;*L. sativa*	Transient ^b^	[157]
*tEU* *–tTM6MAR*	*N. tabacum*	1,931	~23× higher GFP than with *tNOS*;~10× higher DsRed than with *tNOS*	*N. benthamiana*	Transient ^b^	[156]
*tEU (intronless)*	*N. tabacum*	480	~10× higher GFP than with *tNOS*	*L. sativa*	Transient ^b^	[156,158]
			11.9× higher GFP than with *tVspB3*;	*N. benthamiana*		
			2.8× higher GFP than with *t35S*;			
			~15× higher DsRed than with *tNOS*			
*tEU (intronless)* *–tACT3* *–tRB7MAR*	*N. tabacum (tEU; tRB7); N. benthamiana (tACT3)*	2,693	~56× higher GFP than with *tNOS*	*N. benthamiana*	Transient ^b^	[156]
*tHSP18*	*A. thaliana*	249	6.5× or 8.4× higher miraculin protein than with *tNOS*	*S. lycopersicum*	Stable ^c^	[159,160]
		249	4× higher miraculin protein (*SlE8* promoter) than with *MIR-tNOS*	*S. lycopersicum*	Stable ^c^	
*tHSP18*	*A. thaliana*	N.A.	1.5× or 2.5× higher GUS or Fluc than with *t35S*, *tOCS* or *tNOS*	*A. thaliana; O. sativa*	Transient (protoplasts)	[152]
		878	1.5× higher Rluc or Fluc than with 250 bp *tHSP18*	*N. benthamiana*	Stable ^c^	[161]
		249	~7.5× higher GFP than with *tNOS*	*L. sativa*	Transient ^b^	[156]
		249	2.5× higher GFP than with *tNOS*	*N. benthamiana*		
*tHSP18* *–tEU* *–tRb7MAR*	*A. thaliana (tHSP18); N. tabacum (tEU; tRb7)*	1,898	~20× higher GFP than with *tNOS*	*N. benthamiana*	Transient ^b^	[156]
*tHSP18* *–tACT3*	*N. benthamiana (tHSP18; tACT3); N. tabacum (tTM6; tRb7); S. tuberosum (tPINII)*	1,485	~25× higher GFP than with *tNOS*	*N. benthamiana*	Transient ^b^	[156]
*tHSP18* *–tACT3* *–tRb7*		2,654	~50× higher GFP than with *tNOS*			
*tHSP18* *–tPINII* *–tRb7MAR*		2,580	~15× higher GFP than with *tNOS*			
*tHSP18* *–tPINII* *–tTM6MAR*		2,610	~15× higher GFP than with *tNOS*			
*tHSP18* *–tRb7MAR*		1,610	~14× higher GFP than with *tNOS*	*N. benthamiana*	Transient ^b^	[156]
*tProteinase inhibitor II (tPINII)*	*S. tuberosum*	N.A.	20× higher HBsAg than with *tNOS*	*S. tuberosum*	Stable ^c^	[162]
		970	8.5× higher GFP than with *tNOS*	*N. benthamiana*	Transient ^b^	[156]
*trbcS*	*M. domestica*	582	11.1× higher GUS than with *tNOS* (*M. domestica rbcS* promoter – 1,679 bp)	*N. benthamiana*	Transient ^b^	[163]
	*Medicago sativa*	400	~1.4× higher GUS than with *tNOS* (Figwort mosaic virus *35S* promoter)	*M. sativa*	Stable ^c^	[164]
	*Pisum sativum*	684	5.4× higher GFP than with *tNOS*; ~10× higher DsRed than with *tNOS*	*N. benthamiana*	Transient ^b^	[156]

^a^ The *35S* promoter was used except when specified. ^b^ Transient, leaf agroinfiltration. ^c^ Stable, *Agrobacterium*-mediated transformation.

Plant terminators determine which PASs the gene’s mRNA is processed at. For example, Yang et al. [167] overexpressed a modified house dust mite allergen gene *mDerf2* in transgenic rice using the maize *Ubiquitin* promoter together with different terminators and examined where the transgene mRNA was processed in the seeds and leaves of the transgenic rice plants. They found that the transgene mRNA with the rice *glutelin B-1* terminator (*tGluB-1*) was processed at 2 specific PAS sites in the seed but at 6 sites in the leaf. The 2 PAS sites in the seed were identical to those in the native *GluB-1* mRNA. However, when regulated by *tNos*, the transgene mRNA was processed at 8 PAS sites in the seed and at 6 sites in the leaf.

Plant terminators also affect the length of read-through mRNA transcripts, which is a normal phenomenon of gene transcription. Xing et al. [168] studied the effects of soybean native terminators on transgene expression in transgenic soybean plants. They found that read-through transcription occurred in all the transgenic plants, with ~1% of total transgene mRNA being generated from this process. In addition, Hiwasa-Tanase et al. [165] observed longer read-through mRNA transcripts when the transgene was processed by *tNOS* rather than *tMIR*.

Interestingly, studies showed that 2 terminators can be linked together for enhanced transgene expression; perhaps the addition of a second terminator can lead to better detection of read-through transcription and potentially inhibit it [169]. Diamos and Mason [155] combined the *Nicotiana benthamiana* extensin terminator (*tEU*) with the terminator of a tobacco *Actin* (*NbACT3*) gene to regulate *35S:GFP* expression. Transient expression of the reporter gene was observed at levels of 37.7 times greater when the combined terminators were used over *tNos.* Similarly, Yamamoto et al. [170] measured transient *GFP* expression in lettuce *(Lactuca sativa)*, tomatoes, eggplants *(Solanum melongena)*, hot peppers *(Capsicum frutescens)*, melons *(Cucumis melo)*, orchids *(Phalaenopsis aphrodite)*, and tobacco by using combined terminators. Combination of *Arabidopsis tHSP18* and tobacco *tEU* rendered the highest expression when compared to each terminator by itself or a double terminator of the same kind. The combined *tHSP18-tEU* terminator also worked better than a triple terminator made up of one *tHSP*, one *t35S*, and one *tEU*.

The presence of a matrix attachment region (MAR) in a terminator enhances gene expression. MARs are AT-rich DNA sequences that act as epigenetic regulatory sequences by mediating the binding of the chromatin to a protein nuclear matrix and changing chromatin conformation, leading to enhanced gene expression [171]. Matsui et al. [161] compared transient reporter gene expression in tobacco protoplasts when an 878-bp version versus a 250-bp version of the *tHSP18* was used. Higher protein abundance of the *Rluc* and *Fluc* reporter genes was detected when the longer version was used due to the presence of a MAR in the longer version. It was reported that adding MARs into terminators enhanced *GFP* mRNA accumulation and protein production in *Agrobacterium*-infiltrated tobacco leaves [155,158].

Rosenthal et al. [158] tested the effects of the tobacco *tEU* with and without an intron in *tEU* on transient *35S:GFP* expression in tobacco leaves. They found that the intronless *tEU* significantly increased transient *35S:GFP* expression when compared to the intron-containing *tEU*. de Felippes et al. [154] also found that introns located close to the terminator can lead to aberrant termination and splicing in a terminator-dependent manner. They found that having a well-defined poly(A) site increased the likelihood that the transcripts were cleaved at the same site, and inefficient termination leads to transgene silencing. Thus, terminators have a great effect on transgene silencing because they affect siRNA production and splicing efficiency.

## Utilization of Promoters and Terminators for Plant Biodesign

**Fig. 3.**
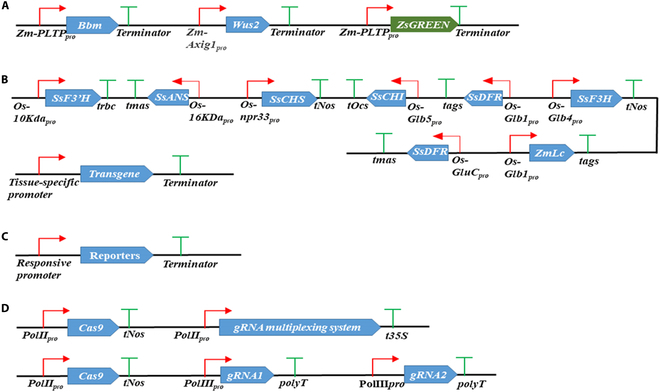
Illustration of construct design for application of promoters in plants. (A) Illustration of the use of promoters for improving plant transformation [174]. (B) Illustration of the use of promoters for metabolic engineering in plants [180]. (C) Illustration of the use of promoters for engineering biosensors in plants. (D) Illustration of the use of promoters for CRISPR/Cas9-based genome editing in plants [185]. *Zm-PLTP_pro_*, maize phospholipid transfer protein gene promoter; *Zm-Axig1_pro_*, maize auxin-inducible *AXIG1* promoter; *Os-10Kda_pro_*, rice 10-kDa prolamin promoter; *trbc*, tobacco *RuBisCO* terminator; *tmas*, mannopine synthase gene terminator from *A. tumefaciens*; *Os-npr33_pro_*, rice 13-kDa prolamin promoter; *tNos*, nopaline synthase terminator from *A. tumefaciens*; *tOcs*, octopine synthase terminator from *A. tumefaciens*; *Os-Glb5_pro_*, rice globulin 5 promoter; *tags*, agropine synthase terminator from *A. tumefaciens*; *Os-Glb4_pro_*, rice globulin 4 promoter; *Os-Glb1_pro_*, rice globulin 1 promoter; *Os-GluC_pro_*, rice glutelin C promoter; *PolII_pro_*, polymerase II promoter; *t35S*, 35S terminator; *PolIII_pro_*, polymerase III promoter; *polyT*, the poly T termination signal for Pol III transcription.

Promoters control the strength and spatiotemporal expression of a gene, while the terminators mainly contribute to the expression level of a gene [19,154,172]. Based on expression patterns, promoters can be categorized into constitutive promoters, responsive/inducible promoters, and tissue-specific (or cell type-specific) promoters (Table 2). With the advent of plant synthetic biology, elegant genetic circuits are needed for fine-tuning gene expression and high-precision genome engineering in plants. Hence, promoters with various strengths and specific expression patterns are necessary for bioengineering of complex plant systems. In this section, we discuss how promoters have been utilized for plant biodesign, with a focus on some representative examples such as plant transformation, plant metabolic engineering, plant-based biosensing, and genome editing (Fig. 3). Through these cases, we cover multiple technical aspects including expression strength, expression patterns, and terminator and promoter configuration.

### Application of promoters for improving plant transformation

Tissue culture-based plant transformation is a rate-limiting step for plant biodesign. Since 2016, morphogenic regulators have been applied to transform recalcitrant cultivars, increase transformation efficiency, shorten the time for plant regeneration, and generate desirable phenotypes while bypassing the tissue culture process [173–175]. Constitutive expression of the maize morphogenic regulators *Baby Boom* (*BBM*) and *Wuschel2* (*WUS2*) promoted direct somatic embryogenesis and thereby increased transformation efficiency in maize [173]. However, this overexpression approach caused side effects, e.g., phenotypic abnormalities and sterility [173], which could be minimized by using tissue-specific or inducible expression of the genes. When the maize leaf- and embryo-specific phospholipid transferase protein (*PLTP*) gene promoter was used to confer a strong tissue-specific expression of *BBM* and the maize auxin-inducible *AXIG1* promoter was used to drive *WUS2* expression, normal transgenic plants were obtained [174] (Fig. 3A).

### Application of promoters for metabolic engineering in plants

Expression of partial or complete biosynthetic pathways from one species can be used for plant metabolic engineering in another species. Multiple genes can be stacked into a plant genome via the use of a single T-DNA sequence. For example, a fungal caffeic acid biosynthesis and recycling pathway containing 4 fungal genes plus a *LUC* reporter gene, with each gene in a constitutive expression cassette, has been used to generate luciferase-based autoluminescent tobacco, tomato, *Arabidopsis*, periwinkle *(Catharanthus roseus)*, petunia *(Petunia hybrida)*, and rose *(Rosa rubiginosa)* plants [176,177]. The stability of transgene expression is always a concern for plant metabolic engineering when the identical expression cassettes are used for the expression of multiple genes [178]. Thus, different promoters are preferred to be used in a multi-gene construct for the expression of different genes [179]. For example, 8 different rice endosperm-specific promoters have been used to drive the expression of 8 anthocyanin biosynthesis pathway genes individually to develop purple endosperm rice [180] (Fig. 3B).

### Application of promoters for engineering biosensors in plants

Precise control of gene expression is essential for plant responses to various environmental stimuli. Stimulus-responsive promoters ensure accurate timing of gene expression. They can be used to drive the expression of optical (fluorescent proteins/luciferases) or morphological reporter genes to form stimulus-responsive “promoter–reporter” systems (Fig. 3C). Whenever a stimulus signal is present, the reporter gene’s expression is activated by the stimulus-responsive promoter, illuminating the dynamics of the signal in real time. Hence, stimulus-responsive promoters are very useful components in plant biodesign for building transcriptional regulation-based plant biosensors. Several phytohormone-responsive promoters have been generated for monitoring phytohormone signaling pathways. For example, the auxin-responsive *DR5* promoter has been used to build biosensors monitoring auxin levels at various developmental stages of *Arabidopsis* roots [181]. In addition, the abscisic acid (ABA)-responsive *DR29* promoter, the cytokinin-responsive *TCS* promoter, and the salicylic acid (SA)-responsive *FLS2* promoter have been used to drive the expression of optical reporters for monitoring the dynamic changes in the levels of their corresponding phytohormones [182–184]. With the availability of rich omics resources, novel *cis*-regulatory elements can be identified and used to generate synthetic stimulus-responsive promoters by multimerizing *cis*-regulatory elements upstream of a core promoter sequence [134–136].

### Application of promoters for CRISPR/Cas9-based genome editing in plants

CRISPR/Cas9-based genome engineering tools are important for plant synthetic biology. There are 2 functional components in the CRISPR/*Cas9* system. The short guide RNA (gRNA) determines the target specificity, while the Cas9 endonuclease generates a double-strand break at each target region [185]. These 2 functional components are usually driven by different expression cassettes. The *Cas9* gene is often driven by a strong constitutive promoter, such as the *35S*, *AtUbi10*, and *ZmUbi* promoters, while the gRNA is usually under the control of a RNA polymerase III (Pol III) promoter such as the *U3* or *U6* promoter [185–187] (Fig. 3D). One limitation of using Pol III promoters is that a specific nucleotide is required in the first position of each transcript, e.g., the *U3* and *U6* promoters in plants have discrete TSSs that are adenine (A) and guanine (G), respectively [188]. Compared with Pol III promoters, Pol II promoters can also be used to express a polycistronic gRNA array, which can be processed posttranscriptionally into individual gRNAs by RNA-cleaving enzymes [189,190], providing a flexibility for spatiotemporal control of multiplexed gene editing.

Tissue-specific promoters can be used to improve the efficiency of genome editing in plants. The *Arabidopsis* meristematic tissue-specific *YAO*, *Egg Cell 1* (*EC1*), and *CLAVATA3* (*CLV3*) promoters have been used to drive *Cas9* expression in *Arabidopsis*, resulting in enhanced CRISPR/*Cas9*-induced mutation efficiency [191]. Similarly, the *Arabidopsis* root cap-specific *SOMBRERO* (*SMB*) promoter, stomatal lineage-specific *TOO MANY MOUTHS* (*TMM*) and *FAMA* promoters, and lateral root primordia-specific *GATA23* promoter have been utilized to drive root cap-, stomatal lineage-, and lateral root primordia-specific knockouts in *Arabidopsis* [192,193].

### Application of combined promoter-terminator combinations for genetic engineering in plants

Promoters can be used together with terminators to fine-tune transgene expression. For example, a total of 105 promoter–terminator combinations have been evaluated in *N. benthamiana* leaves and *N. tabacum* cells, providing a wide range of expression strength [172]. Moreover, Schaart et al. [163] studied the effects of the promoter–terminator combinations on the β-glucuronidase (*GUS*) reporter gene expression by using the *35S* promoter, 2 variants (with different lengths) of the apple (*Malus* × *domestica*) *RbcS* (*MdRbcS*) promoter, and the *tNos* and *MdRbcS* terminator (*tMdRbcs*). Using transient expression in tobacco leaves, they found that the *35S* promoter*–tMdRbcs* combination generated the highest *GUS* mRNA and protein levels, followed by the *35S* promoter*–tNOS*, and each *MdRbcS* promoter*–tMdRbcs*. The lowest *GUS* expression was rendered by the *MdRbcS* promoter*–tNos* combination. Similarly, Kurokawa et al. [160] found that the transgenic tomato plants expressing the *MIR* transgene by the tomato fruit-ripening specific *E8* promoter*–Arabidopsis tHSP18* produced 4 times more MIR protein per fruit fresh weight than the transgenic tomato plants containing *35S:MIR–tNos*. Thus, it is important to select an appropriate promoter–terminator combination for optimal transgene expression in plant biodesign [172].

## Strategies for Benchmarking Promoters and Terminators for Plant Biodesign

To date, more than 8,000 plant promoter sequences have been identified through transcriptomic and genomic analyses and documented in various plant promoter databases such as PlantProm DB [117], PlantPromoterDB [119], and PlantCARE [129]. However, only a small number of promoters and terminators have been experimentally characterized and validated in plants (see above). Thus, there is high demand to expand the number of functionally characterized promoters and terminators to serve as standard biological parts for plant synthetic biology research and bioengineering. Benchmarking via standardized procedures is an essential step for providing necessary semiquantitative and quantitative information about the performance of promoters and terminators.

### Requirements for evaluating promoters and terminators in plants

**Fig. 4.**
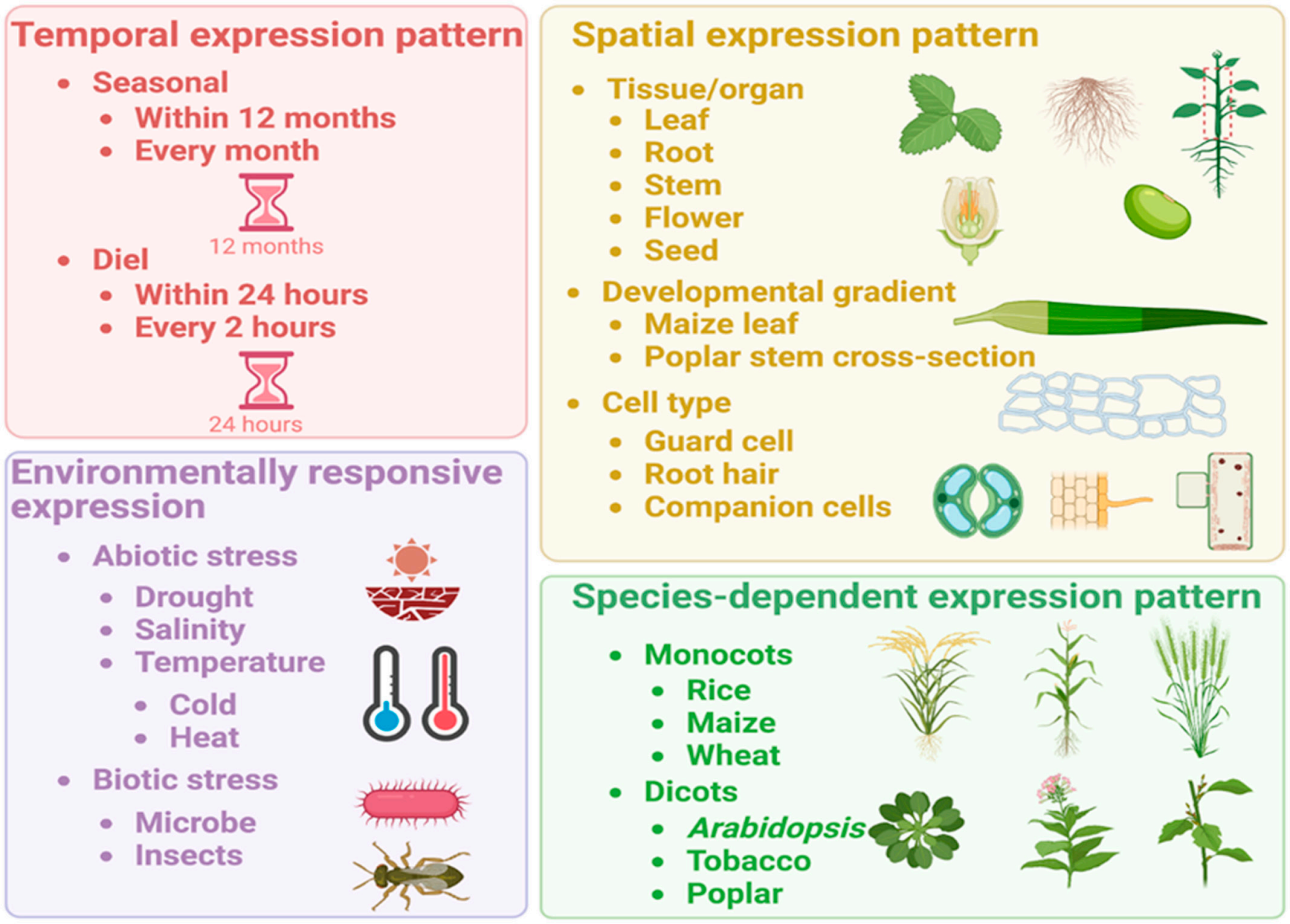
Aspects of promoter and terminator evaluation for plant biodesign.

The features and performance of promoters and terminators can be systematically evaluated in 4 aspects: (a) temporal expression pattern, (b) spatial expression pattern, (c) environmental responses, and (d) cross-species variation (Fig. 4). For each of the 4 aspects, the strength of promoters and promoter–terminator combinations needs to be quantitatively evaluated. Promoters can be explored based on seasonal and diurnal patterns. In terms of spatial expression patterns, numerous tissue- and cell type-specific promoters have been identified in plants including leaf-, stem-, root-, flower-, fruit-, guard cell-, root hair-, and companion cell-specific promoters [194]. Developmental gradient is also one of the aspects that reflect the spatial expression patterns of some promoters. For instance, the developmental gradient in maize leaves has been exploited to redefine the current C_4_ model and gain new insights into the regulation of C_4_ photosynthesis [195]. Notably, the performance and the relative usefulness of individual promoters and terminators may be different across plant species. It would be necessary to characterize promoters and terminators in different plant species representing dicots (e.g., *Arabidopsis*, tobacco, poplar) and/or monocots (e.g., rice, maize, wheat *(Triticum aestivum)*). Bioengineering of stress tolerance in plants requires the identification and characterization of environmental stress-responsive promoters [196], which can be tested under biotic treatments (e.g., pathogens and beneficial microbes) and abiotic stresses (e.g., drought, salinity, and temperature).

### Methodologies for benchmarking promoters and terminators in plants

**Fig. 5.**
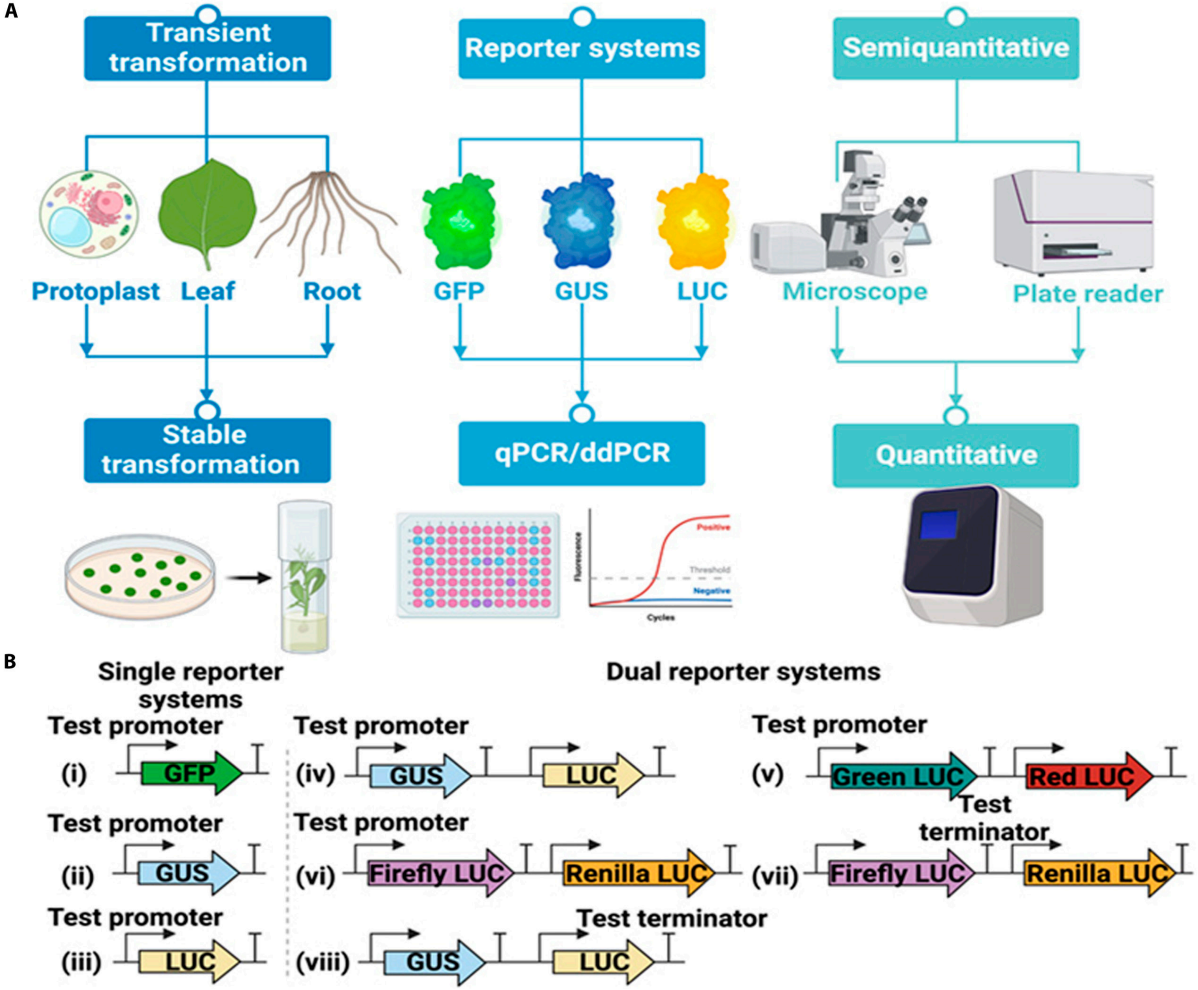
The technologies for benchmarking promoters and terminator in plants. (A) Methods and tools used for performance analysis of plant promoters and terminators. (B) Different architectures of single-reporter and dual-reporter systems. ddPCR, droplet digital PCR; LUC, luciferase.

Many different methodologies for validating plant promoters and terminators have been developed and applied to various plant species. These methodologies can be classified into 2 main categories, i.e., transient expression approaches and stable expression approaches (Fig. 5A). Transient expression approaches are relatively simple and effective and can be quickly completed in various plant cells and tissue/organs. Examples for transient expression systems include protoplast transfection, leaf agro-infiltration, and hairy root transformation. In contrast, stable expression systems involve complex and lengthy stable plant transformation but provide the most robust information on the function and strength of promoters and terminators. Reporter genes such as *GFP*, *GUS*, and *LUC* are often used to pinpoint the performance of a target promoter and/or terminator in either transient or stable expression systems (Fig. 5A). The reporter assay for the analysis of promoter and/or terminator activity in a transient expression system allows for semiquantitative determination of reporter gene expression using microscope and/or plate reader, leading to variable output due to the use of different cell types and sample preparation methods. Reverse transcription quantitative polymerase chain reaction (RT-qPCR) and droplet digital PCR (ddPCR) analysis of transgenic plant samples can provide more reliable and robust information on promoter and terminator features (Fig. 5B). Moreover, certain features (e.g., environmental response or time-course development) of a target promoter and/or terminator can only be investigated using stable transformed plants or samples.

Both single-reporter and dual-reporter systems have been used to examine promoter and terminator performance in either transient or stable expression systems. A single-reporter system allows reporter gene expression to be imaged and detected in the samples of interest through a microscope and/or a plate reader when compared to the samples of mock treatments (Fig. 5A and B). A dual-reporter system provides the means for normalization of the reporter gene expression when a weak promoter–terminator combination is used to drive the expression of a second reporter gene that serves as a reliable internal standard for normalization. The use of a dual-reporter system permits the elimination of the difference in delivery efficiency (for transient expression systems) or positional effects of the transgene (for stable expression systems). To date, multiple dual-reporter systems have been developed for the quantitative characterization of plant-based promoters (Fig. 5B). For example, a GUS/LUC system, whereby LUC was used to normalize and calculate the relative GUS activities, was used to generate relative promoter activities in wheat protoplasts [197]. A dual-color luciferase ratiometric reporter system with green- and red-emitting luciferases was developed for fast characterization of transcriptional regulatory elements in plants [198]. However, the partial signal overlap between green/LUC and red/LUC hinders the highly precise evaluation of the genetic parts. For more precise promoter characterization, the FLUC/RLUC luciferase reporter system is more sensitive since the assay is based on a chemiluminescence reaction and has markedly reduced interference between the 2 luciferases [172]. The FLUC/RLUC assay was used to examine the strength of combinations of promoters and terminators, revealing a 326-fold difference in the level of reporter gene expression between the strongest and the weakest promoters tested in plants [172]. Using a GUS/LUC reporter system, it was reported that appropriate selection of terminator sequences is an important factor for transgene expression in both monocot and dicot plants [199].

## Conclusion and Perspectives

Gene expression is largely under the control of promoters and terminators. Although the selection of promoters and terminators is important for plant bioengineering, efforts to conduct comprehensive sequence analysis of promoters, promoter motifs, and terminators remain limited, hindering high-precision plant bioengineering. The traditional means of identifying regulatory elements in the upstream, intronic, and downstream sequences of genes of interest have limitations. For instance, promoter lengths are frequently chosen in an arbitrary manner (e.g., 1 or 2 kb from the start codons or the TSSs) and regulatory elements at distal sites are sometimes missed. Sequencing-based approaches now permit precise determination of TSSs by mapping nascent transcripts [200]. A recent study used template-switching reverse transcription in conjunction with rolling circle amplification (Smart-Seq2 Rolling Circle to Concatemeric Consensus, or Smar2C2) for global mapping of TSSs in multiple plant species [201]. The growing adoption of ATAC-seq (assay for transposase-accessible chromatin using sequencing) provides a rich resource for mining the regulatory landscape in open chromatin regions, which are genomic sites accessible by TFs. ATAC-seq analysis across multiple plant genomes revealed that the majority of accessible regions fall within 3 kb upstream of the TSSs [202,203]. Those studies also uncovered a substantial number of distal *cis*-regulatory elements. For instance, more than 10% of ATAC-seq signals are located at >20 kb away from their nearest genes in maize [204]. Integration of RNA-seq and ATAC-seq across a wide range of tissues and increasingly with single-cell resolution should facilitate discovery of tissue-specific *cis*-regulatory elements for precision gene manipulation. Benchmarking *cis*-regulatory elements, promoters, and terminators with different expression patterns and strength will provide invaluable insight for characterizing, curating, and constructing genetic circuits and pathways in plant biodesign.

The orthogonality of the key regulatory elements present in the promoters and terminators permits the use of these biobricks in a heterologous system for plant biodesign. However, these regulatory elements (as well as the promoters and terminators themselves) are optimized to function properly in their native contexts, and their regulation in a heterologous system may be different from that in their natural contexts [205]. Thus, whether these biobricks function in an orthogonal manner in a different species must be taken into consideration. Combining a trial-and-error approach with fine-tuning for design, testing, and validation will help obtain the desired functions and properties [1].
